# Recent Bioengineering Strategies and Clinical Applications in Dentistry of Oral and Maxillofacial Organoids

**DOI:** 10.1016/j.identj.2026.109777

**Published:** 2026-08-01

**Authors:** Mahtab Mottaghi, Amir Attaran Khorasani, Shahrzad Samadian, Alieh Farshbaf, Mehran Mohareri, Farzaneh Ahrari

**Affiliations:** a School of Dentistry, Mashhad University of Medical Sciences, Mashhad, Iran; b Department of Pediatric Dentistry, School of Dentistry, Shahid Beheshti University of Medical Sciences, Tehran, Iran; c Dental Research Center, Mashhad University of Medical Sciences, Mashhad, Iran; d Immunology Research Center, Inflammation and Inflammatory Diseases Division, Mashhad University of Medical Sciences, Mashhad, Iran

**Keywords:** Organoids, Oral and maxillofacial diseases, Tissue engineering, Regeneration, Precision medicine

## Abstract

**Introduction and aims:**

The complex, interconnected nature of dental, oral, and craniofacial tissues poses significant challenges for conventional *in vitro* models. Oral and maxillofacial organoids have emerged as advanced three-dimensional technologies capable of reproducing selected microstructures and biological functions of native tissues. This review aimed to summarize recent advances in these technologies and evaluate their potential clinical applications in dentistry.

**Methods:**

A narrative literature review was conducted using PubMed, Web of Science, Scopus, and Google Scholar, with particular focus on studies published between 2020 and 2026. The search terms included ‘organoids’, ‘oral and maxillofacial diseases’, ‘tissue engineering’, ‘regeneration’, and ‘precision medicine’. Relevant studies addressing organoid development, bioengineering strategies, and clinical applications in dentistry were analysed.

**Results:**

Recent advances have enabled the development of various oral and maxillofacial organoids, including tooth-germ, salivary gland, taste bud, oral cancer, lingual epithelial, and maxillofacial cartilage organoids. Advances in patient-derived organoids through construction strategies, such as spontaneous self-assembly of stem cells, biomaterial-assisted fabrication, and precision engineering, enhance their physiological properties and support personalized medicine. Despite these advances, several challenges remain that restrict clinical translation, including limited vascularization, lack of immune system integration, structural variability, scalability, and standardization issues.

**Conclusion:**

Oral and maxillofacial organoids represent promising experimental platforms for regenerative dentistry, disease modelling, drug screening, and tissue regeneration. Combining organoids with microfluidic technologies to create organ-on-a-chip devices and linking multiple chips represents important advances towards the reliable, large-scale generation of physiologically relevant oral and maxillofacial organoids for both research and clinical applications.

**Clinical relevance:**

Oral organoid systems may provide clinical platforms for personalized therapy, regenerative applications, and translational research while reducing reliance on conventional animal models. Among current models, oral cancer and salivary gland organoids demonstrate promising translational potential, given their functional validation and scalability.

## Highlights

• Oral and maxillofacial organoids are developed for disease modelling, drug screening, tissue repair and regeneration, and the oral microbiome.
• Organoid platforms reduce dependence on conventional animal models.
• Vascularization, immune integration, scalability, and standardization are main barriers in clinical applications of organoids.
• Among current models, oral cancer and salivary gland organoids demonstrate promising translational potential due to their functional validation and scalability.
• Bridging the gap between experimental models and clinical applications will require further research in the future.

## Graphical abstract

**Figure:**
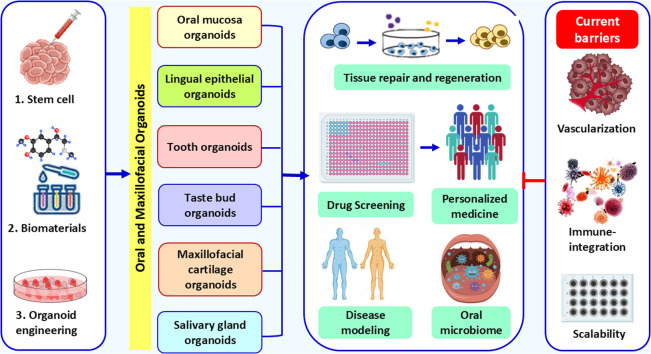

## Introduction

**Fig. 1:**
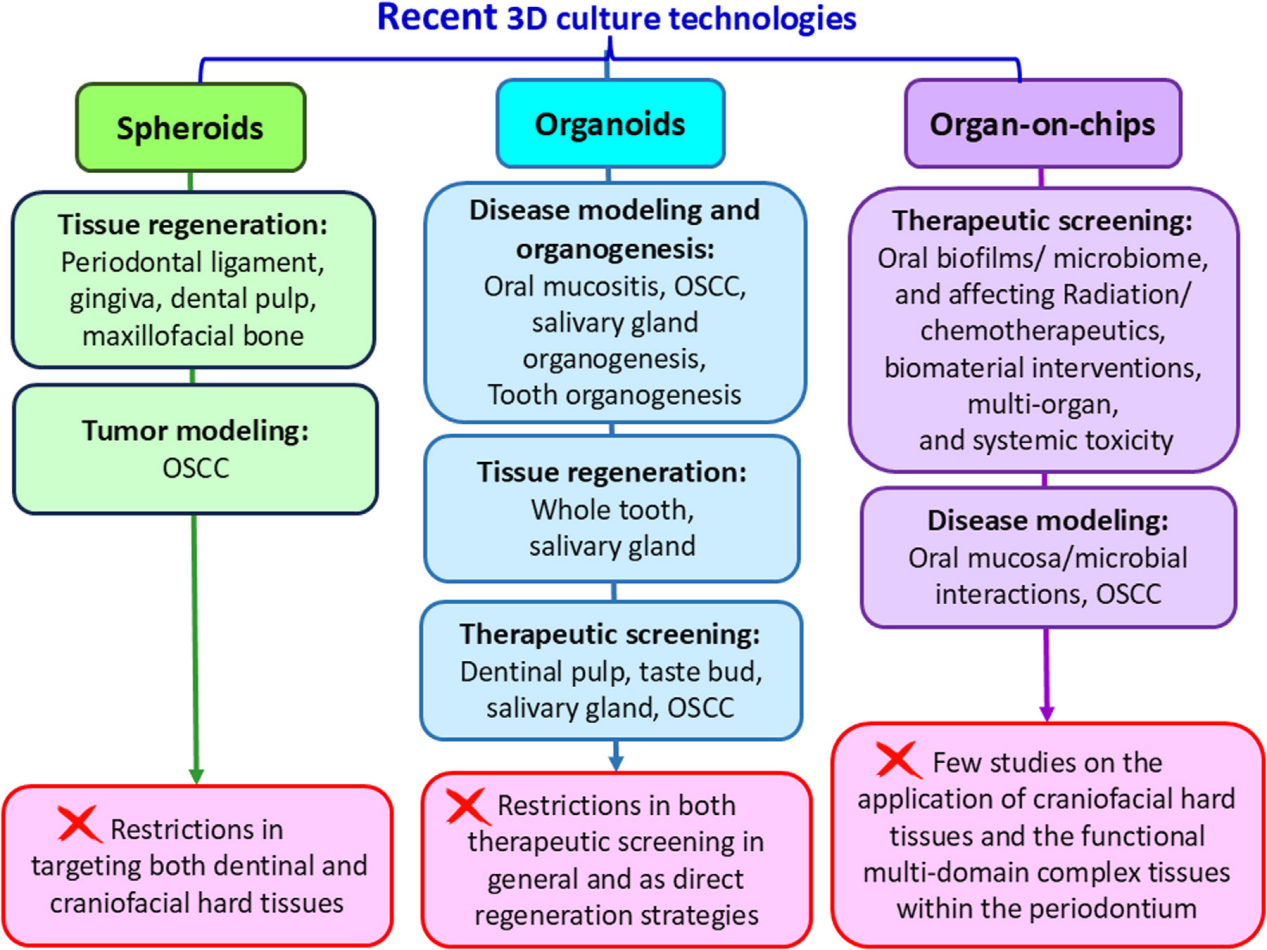
Overview of the major applications and current limitations of advanced three-dimensional (3D) spheroid, organoid, and organ-on-a-chip (OoC) systems in oral and maxillofacial research. These platforms support disease modelling, regenerative dentistry, drug screening, precision medicine, and oral microbiome studies under physiologically relevant conditions. The figure also highlights key clinical challenges, including limited vascularization, insufficient immune integration, structural heterogeneity, scalability constraints, and difficulties in standardization. OSCC, oral squamous cell carcinoma.

The complex and interconnected nature of dental, oral, and craniofacial tissues makes it challenging to reproduce their defects and diseases using conventional two-dimensional (2D) and 3D *in vitro* culture models. Recent advances in 3D culture technologies, including spheroids, organoids, and organ-on-a-chip (OoC) technologies, reproduce important structural and functional characteristics of clinically relevant disease states (Figure 1).^1^ Among them, organoids are advanced 3D *in vitro* models that maintain the genetic and phenotypic characteristics of native human tissues. They have been shown to recapitulate selected features of native microstructures, originating from normal or tumour tissues. When generated from patient-derived cells, patient-derived organoids can model various diseases, including oral and maxillofacial diseases, tumours, and developmental dental abnormalities. These properties make them a useful experimental system for investigating oral and maxillofacial tissue development, pathology, and regeneration *in vitro*.^2^ Organoids have also demonstrated improved differentiation into diverse progenitor cell populations, supporting the development of organ-specific tissue models.^3^^,^^4^ Moreover, organoid cultures can be maintained long-term and effectively cryopreserved, exhibiting greater genomic and transcriptomic stability than progenitor cells, which are prone to high mutation rates. As extended oral and maxillofacial organoids, these microphysiological systems are increasingly employed for drug screening to evaluate both therapeutic efficacy and toxicity.^5^

This narrative review primarily evaluated studies published between 2020 and 2026 through searches of the Web of Science, PubMed, Google Scholar, and Scopus databases. The searching keywords included ‘organoids’, ‘oral and maxillofacial diseases’, ‘tissue engineering’, ‘regeneration’, and ‘precision medicine’, combined using the Boolean operators ‘AND’ and/or ‘OR’. Relevant studies addressing organoid development, bioengineering strategies, and clinical applications in dentistry were analysed.

The present study focused on the comparative clinical status of oral and maxillofacial organoid models and current unmet clinical needs in dentistry. In addition to summarizing recent advances in organoid bioengineering, coculture strategies, and organoid-on-chip integration, we emphasized potential roles of oral and maxillofacial organoids in regenerative dentistry, disease modelling, personalized medicine, and oral oncology. Furthermore, we discussed the main translational barriers, including vascularization, immune integration, scalability, and standardization, which remain critical limitations for future clinical implementation and present potential solutions.

## Development of different oral and maxillofacial organoids

Different types of oral and maxillofacial organoids have been developed to study oral cavity diseases, drug screening, and the evaluation of cell behaviour in response to environmental conditions. In Table, we summarized potential applications of oral and maxillofacial organoids based on their cell sources for disease modelling, along with their benefits and restrictions.

**Table:** A summary of clinical applications of oral and maxillofacial organoids.

Organoid	Source	Function	Disease modelling	Advantages	Limitations
Salivary gland organoid	Adult human salivary gland stem/progenitor cells	Used for treating radiation-induced xerostomia and as a strategy to restore salivary gland function	Sialadenitis, Radioactive salivatitis, xerostomia/Sjögren syndrome, SG tumour, SG defects, and regeneration	Capable of generating salivary gland-like structures with normal functional features	Lacks vascularization; organoids remain small in size
Tooth-germ organoid	Adult human dental pulp cells	Enables investigation of fundamental mechanisms in tooth development	Pulpitis, apical periodontitis, periodontitis, tooth loss, and regenerative applications	Formation of tooth-like constructs with complete structure and vascularization	Difficult to control and maintain uniform tooth morphology
Lingual epithelial organoid	Bmi1^+^ cells isolated from the basal layer of mouse lingual epithelium	Supports studies of lingual epithelium biology, regeneration, and carcinogenesis	Tongue tumour, glossitis, oral mucositis, tongue defects, and regeneration	Growing and maturing in the muscular layer of the tongue instead of only in the epithelial layer	Lack of components that mimic the *in vivo* niche and control self-organization to form a specific morphology
Taste bud organoid	Lgr5^+^ stem cells from murine circumvallate papillae	Used to study taste signalling pathways, disease mechanisms, and taste tissue regeneration	Taste dysfunction, chemotherapy-induced taste loss, and inflammatory taste disorders	Organoids can reproduce key aspects of taste epithelium homeostasis	Cannot dynamically model real-time taste signal transmission
Mucosal organoid	Primary OSCC cells; primary normal keratinocytes	Supports expansion of drug-testing platforms and personalized therapy for head and neck cancers	Viral infections, oral premalignant lesions, mucosal tumours (OSCC)	Demonstrates migration and invasion patterns similar to OSCC	Cannot fully replicate the complexity of the tumour microenvironment
Maxillofacial cartilage organoids	Neural crest stem cells	Enables investigation of cartilage formation and potential regenerative applications	Craniofacial cartilage defects, trauma-related cartilage loss, and congenital abnormalities	Organoids show robust expression of cartilage differentiation markers	Effective methods to regenerate maxillofacial cartilage with complete structure and mechanical strength remain undeveloped

HNSCC, head and neck squamous cell carcinoma; OSCC, oral squamous cell carcinoma; SG, salivary gland; TMD, temporomandibular disorders.

### Tooth-germ organoids

Tooth organoids are generated through epithelial–mesenchymal interactions supported by collagen-based scaffolds and growth factor supplementation, which promote the formation of tooth-like structures.^6^ The resulting tooth-germ organoids have demonstrated regenerative potential in experimental models after transplantation into alveolar sockets. Similar approaches may contribute to future bioengineered tooth regeneration strategies.^7^^,^^8^

To further bridge the gap between natural and organoid-derived teeth, various bioengineered scaffolds have been developed to enhance tooth-germ organoid formation, such as electrospun poly(lactic-co-glycolic acid) hydrogels, human umbilical vein endothelial cell-based gelatin methacrylate (GelMA) hydrogels, and GelMA hydrogel microparticles.^9^ Nevertheless, the limited availability of autologous tooth germs, as they primarily originate from third molars, limits the generation of anterior teeth due to differences in genetic lineage. This limitation has prompted the use of pluripotent stem cells (PSCs) in developing tooth-germ organoids. For example, the coculture of mouse dental mesenchymal cells and PSCs can form tooth-like structures, which were implanted into alveolar sockets or cultured with electrospun polycaprolactone (PCL) membranes.^10^

Beyond scaffold composition, the culture microenvironment also contributes to organoid development. Tooth-germ organoids coated with electrospun PCL membranes functionalized with various growth factors may partially reproduce aspects of the native microenvironment, facilitating organogenesis and vascularization. These bioactive membranes release biochemicals that support organoid maturation, vascularization, and tissue organization.^11^

### Taste bud organoids

Taste bud organoids provide a valuable *in vitro* model for investigating the mechanisms underlying taste function and for promoting taste bud regeneration. Conventional culture methods, however, fail to achieve proper spatial organization of taste receptor cells. In contrast, a suspension culture combined with Matrigel, which is widely used to promote the survival of various cell types, may recapitulate epithelial homeostasis. Consequently, this approach increases the number of stem cells and taste receptor cells and may facilitate future regenerative applications.^12^ Moreover, decellularized tongue-derived matrices can mimic the native taste bud microenvironment, serving as efficient and sensitive platforms for taste sensing that enhance cell adhesion and provide essential growth factors.^13^

Taste bud organoids have also emerged as an area of active investigation in oral oncology, particularly for patients with taste dysfunction following radiotherapy, chemotherapy, or xerostomia associated with salivary gland hypofunction. These systems provide valuable experimental models for studying taste receptor regeneration, epithelial repair, and treatment-related sensory dysfunction under physiologically relevant conditions.^14^ The development of microfluidic devices can support future clinical applications of taste bud regeneration by providing a stable, standardized system for monitoring taste responses in organoids.^15^

### Salivary gland organoids

Rather than being unique to salivary gland organoids, the combined influence of intrinsic regulatory mechanisms and extrinsic niche-derived signals represents a fundamental principle underlying the establishment, differentiation, and functional maintenance of oral and maxillofacial organoid systems. *In vitro* studies have shown that seeding human salivary gland cells in Matrigel scaffolds can support organoid formation.^16^ These organoids not only exhibit morphologies and gene expression profiles similar to those of native glands but also secrete saliva after transplantation into mice. Nonetheless, the use of nonhuman-derived scaffolds limits their clinical application for human salivary gland repair and regeneration.^17^ To address these challenges, bioengineered salivary gland organoids have been developed using a niche-independent approach involving PEG hydrogel-incorporated PCL nanofibrous microwell scaffolds.^18^ Additionally, hybrid scaffolds combining elastin with poly(lactic-co-glycolic acid) nanofibres have been utilized to enhance the self-organization of epithelial cells.

### Oral cancer organoids

Oral cancer organoids are used to examine tumour cell behaviour in response to varying drug doses, assess drug resistance and toxicity, and detect biomarkers for diagnostic and prognostic purposes. In addition, this technology offers opportunities for single-cell and high-throughput analysis, as well as the study of cancer stem cells (CSCs), opening new avenues for personalized medicine in the field of oral cancer.^19^^,^^20^ Additionally, CSCs isolated from patient samples support the generation of oral cancer organoids through a Matrigel-based culture system that retains selected molecular characteristics and cellular heterogeneity of the original tumours. However, maintaining the enrichment and expansion of CSCs *in vitro* remains challenging. In a study, coculturing cancer-associated fibroblasts with oral cancer organoids showed enhanced stem cell properties and improved organoid formation.^21^

Recent advances in bioengineering have further improved oral cancer organoids’ ability to reflect the tumour microenvironment. For example, decellularized tongue extracellular matrix (ECM) has been applied to develop tongue squamous cell carcinoma (TSCC) organoids.^22^ In parallel, nonwoven silica fibre sheets have been introduced as novel scaffolds for oral squamous cell carcinoma (OSCC) organoids.^23^

### Lingual epithelial organoids

Following transplantation of lingual epithelial organoids, the grafted organoids are expanded and matured within the tongue’s muscular layer, emphasizing their potential for lingual regeneration. Nevertheless, a significant limitation of the approach mentioned is the difficulty of consistently producing organoids with uniform morphology, even under identical culture conditions. This inconsistency may result from inadequate replication of the *in vivo* niche and from uncontrolled cell self-organization during organoid formation.^24^

Current lingual epithelial organoids have demonstrated the capacity to repair both the muscular and epithelial layers of the tongue. However, there remains an urgent need for innovative bioengineering strategies to further improve organoid development. Future studies should aim to regulate stem cell self-organization within organoids by examining complex cellular niches and employing shape-controlled culture systems to reproduce fully functional lingual epithelial organoids that closely resemble native tissue for clinical regeneration.^25^

### Maxillofacial cartilage organoids

Maxillofacial cartilage involves the hyaline cartilage of the nose, developing craniofacial bones, and the fibrocartilage of the temporomandibular joint. Recently, hyaline cartilage organoids encapsulated in alginate hydrogel with appropriate elastic properties have been developed. This system enhanced cell proliferation, indicating high chondrocyte activity after 24 days of *in vitro* culture.^26^ These findings demonstrate the feasibility of recapitulating cartilage/bone organoids with active chondrocytes, suggesting strong potential for large-scale cartilage regeneration.^27^ However, the applicability of this method for human cartilage regeneration remains to be confirmed, as validation using human chondrocytes is still required. Current challenges include the limited availability of human embryonic stem cells and the risk of xenogeneic immune rejection. Therefore, the use of human PSCs and adult stem cells is recommended for developing maxillofacial cartilage organoids suitable for clinical applications.^28^

## Comparison of the clinical potential of oral and maxillofacial organoid models

To provide a more structured comparative evaluation, the potential translation of oral and maxillofacial organoid technologies was assessed based on some parameters, including the degree of structural and functional resemblance to native tissues, reproducibility and scalability of organoid generation, incorporation of physiologically relevant features (vascular or immune components), suitability for disease modelling and drug screening, and overall potential for regenerative dentistry and personalized medicine.^15^^,^^29^ The result showed that the current organoid models demonstrated varying levels of biological maturity and translational potential.

Altogether, tooth-germ organoids represent one of the most advanced systems for recapitulating epithelial-mesenchymal interactions and achieving whole-organ formation. Nonetheless, their reliance on specific cell sources and complex developmental procedures limits their widespread applicability.^8^ In contrast, salivary gland and taste bud organoids have demonstrated capabilities for functional regeneration, especially in conditions such as xerostomia and taste dysfunction. Salivary gland organoids have shown encouraging results in restoring secretory function following transplantation.^30^ Taste bud organoids, while valuable for mechanistic studies, remain limited by insufficient functional validation and standardized response assays.^31^ Oral cancer organoids demonstrate promising translational potential, particularly for drug screening, biomarker detection, and personalized medicine, while preserving tumour heterogeneity and patient-specific characteristics. Their integration with stromal and immune components in culture conditions further enhances their physiological features, positioning them as leading platforms in preclinical oncology research.^20^

Meanwhile, lingual epithelial and maxillofacial cartilage organoids are still in relatively early stages of development. Although they show promise for soft tissue and craniofacial regeneration, issues related to reproducibility, structural organization, and validation in human systems need to be addressed before clinical application can be realized.^19^ Among current models, oral cancer and salivary gland organoids demonstrate promising translational potential, given their functional validation and scalability.

## Construction strategies

The development of physiologically relevant oral and maxillofacial organoids depends on recapitulating selected features of the native tissue microenvironment. Achieving this requires precise control over stem cell differentiation and 3D self-organization through bioengineering strategies. Current approaches for constructing such organoids include: (1) spontaneous self-assembly of stem cells, (2) biomaterial-assisted fabrication, and (3) precision engineering technologies, such as 3D bioprinting and microfluidic technology.^32^

### Spontaneous self-assembly of stem cells

Self-assembly techniques utilize the intrinsic ability of cells to organize into 3D structures, mimicking processes that occur during embryonic development. Through interactions mediated by cell adhesion molecules and the ECM, this scaffold-free approach allows the spontaneous formation of complex tissue constructions. In dental research, self-assembly has emerged as an effective strategy for generating organoids that potentially replicate odontogenic tissues, such as dental pulp and dentin.^33^ The potential of coordinated self-assembly is particularly evident in recreating complex tissue interfaces, including tooth root-like structures. When dental pulp stem cells (DPSCs) are cocultured with periodontal ligament stem cells (PDLSCs) in defined ratios, they spontaneously form bilayer organoids with distinct tissue compartments.^34^ The inner core develops into tubular dentin-like tissue, potentially resembling the natural tooth root interface.^35^

### Biomaterials-assisted construction

Scaffold materials are essential for creating a 3D microenvironment that supports and guides tissue formation. Natural polymers are commonly used due to their high biocompatibility, which preserves native ECM proteins and promotes DPSC migration and mineralization.^36^ GelMA hydrogels have also become prominent materials for dental pulp regeneration due to their injectability, rapid gelation, and excellent biocompatibility.^37^ In addition, synthetic polymers with favourable physical characteristics have been utilized to create hydrogels or nanofibre scaffolds that support the chondrogenic differentiation of DPSCs and facilitate organoid tissue formation.^38^

### Precision engineering technology

The development of organoids derived from dental pulp and periodontal tissues depends on the selection of suitable fabrication strategies. Techniques such as 3D bioprinting and microfluidic systems contribute to recapitulating selected features of the natural architecture and physiological functions of these tissues.

#### 3D bioprinting for precision tissue construction

3D bioprinting reflects the biomimetic fabrication of complex oral tissues through computer-assisted spatial control and layer-by-layer deposition of bioinks. This technology shows great promise for dental pulp regeneration, periodontal reconstruction, and jawbone repair, with recent advances in bioink development and multiscale structural design.^39^^,^^40^ In one study, bioprinted dentin-pulp constructs with instructive niches for regeneration enhanced the viability, migration, and proliferation of embedded DPSCs.^41^ The significant advantage of 3D bioprinting is its capacity to reproduce the hierarchical structure and heterogeneous interfaces of native tissues. Multimaterial printing techniques further enhance this capability by the simultaneous or sequential deposition of bioinks with distinct compositions, thereby preserving a precise model of complex tissue boundaries.^42^

#### Microfluidic technology

Unlike static culture systems, microfluidic platforms may partially reproduce aspects of the dynamic oral environments, such as salivary flow and masticatory forces.^43^ By harnessing the natural processes that enable organoid self-assembly, microfluidic systems provide a highly biomimetic and controllable platform for *in vitro* culture and testing, showing promising potential in dental pulp regeneration, salivary gland modelling, and periodontal tissue engineering.^44^

The primary advantage of microfluidic technology is the spatial coordination of interactions among different cell types and tissue structures. A representative example is the gingival sulcus-on-a-chip model, which integrates keratinocytes, fibroblasts, and endothelial cells within defined microenvironments exposed to continuous perfusion. By closely recapitulating the multilayered epithelial barrier, the model enables physiologically relevant investigations of inflammatory mechanisms and wound-healing dynamics.^45^ Similarly, a ‘tooth-on-a-chip’ model combining dentin discs and DPSCs has been used to examine dentin permeability and DPSC injury following exposure to silver diamine fluoride.^46^ More advanced ‘implant-on-a-chip’ systems integrate titanium surfaces with gingival cocultures, recreating the complex 3D interactions among host tissue, biomaterials, and oral microbiota to study the pathogenesis of peri-implantitis and implant biocompatibility.^47^ In addition, microfluidic platforms that mimic physiological saliva flow have revealed how fluid dynamics shape the cariogenic potential of dental biofilms by influencing local pH distributions and acid accumulation within the biofilm microenvironment.^48^

In dentistry, organ-on-chip platforms may provide more physiologically relevant environments for investigating oral carcinogenesis, salivary gland dysfunction, host-microbiome interactions, and patient-specific therapeutic responses. For instance, a continuous-flow PCR array chip leads to the rapid identification of three major periodontal pathogens within 8 minutes.^49^ Another study demonstrated a strong linear correlation between bacterial concentration and signal output with a microfluidic chip technology. The rapid and quantitative detection of *Streptococcus mutans*, the principal bacterium associated with dental caries, highlights the potential of microfluidics as compact, accurate, and point-of-care diagnostic tools for early caries risk assessment in dentistry.^50^ Additionally, microfluidic drug-screening platforms can model periodontal pocket fluid dynamics, allowing for the optimization of localized antimicrobial and regenerative treatments.^51^

### Other stem cell sources for organoid development

In addition to DPSCs, which are widely utilized due to their strong odontogenic potential and relevance to dental tissues, alternative stem cell sources are increasingly being investigated for oral and maxillofacial organoid development. Induced PSCs (iPSCs) offer a flexible and extensible platform owing to their pluripotency and capacity for patient-specific applications, reproducing the generation of diverse oral cell lineages, including epithelial and mesenchymal components.^52^ An important consideration in the use of iPSCs is the influence of tissue-of-origin effects and residual epigenetic memory, which result in lineage bias by a preferential differentiation tendency towards their tissue of origin. This procedure affected functional heterogeneity among organoids and reduced reproducibility across different iPSC lines.^53^^,^^54^

Another stem cell source is adipose-derived stem cells, being abundant, easily isolated with minimal invasive harvesting procedures. These cells exhibit robust proliferative capacity and multilineage differentiation potential, making them suitable for soft tissue regeneration. Nevertheless, their odontogenic differentiation potential is generally lower than that of DPSCs, which may limit their application in tooth-specific organoid models.^55^

Bone marrow-derived mesenchymal stem cells are another stem cell source that demonstrates promising regenerative capabilities in both hard and soft tissues. They have been successfully applied in craniofacial regeneration and exhibit reliable differentiation into osteogenic and chondrogenic lineages. However, their clinical use is constrained by invasive harvesting procedures, limited cell yield, and interdonor variability.^56^

## Application of oral and maxillofacial organoids in the field of dentistry

**Fig. 2:**
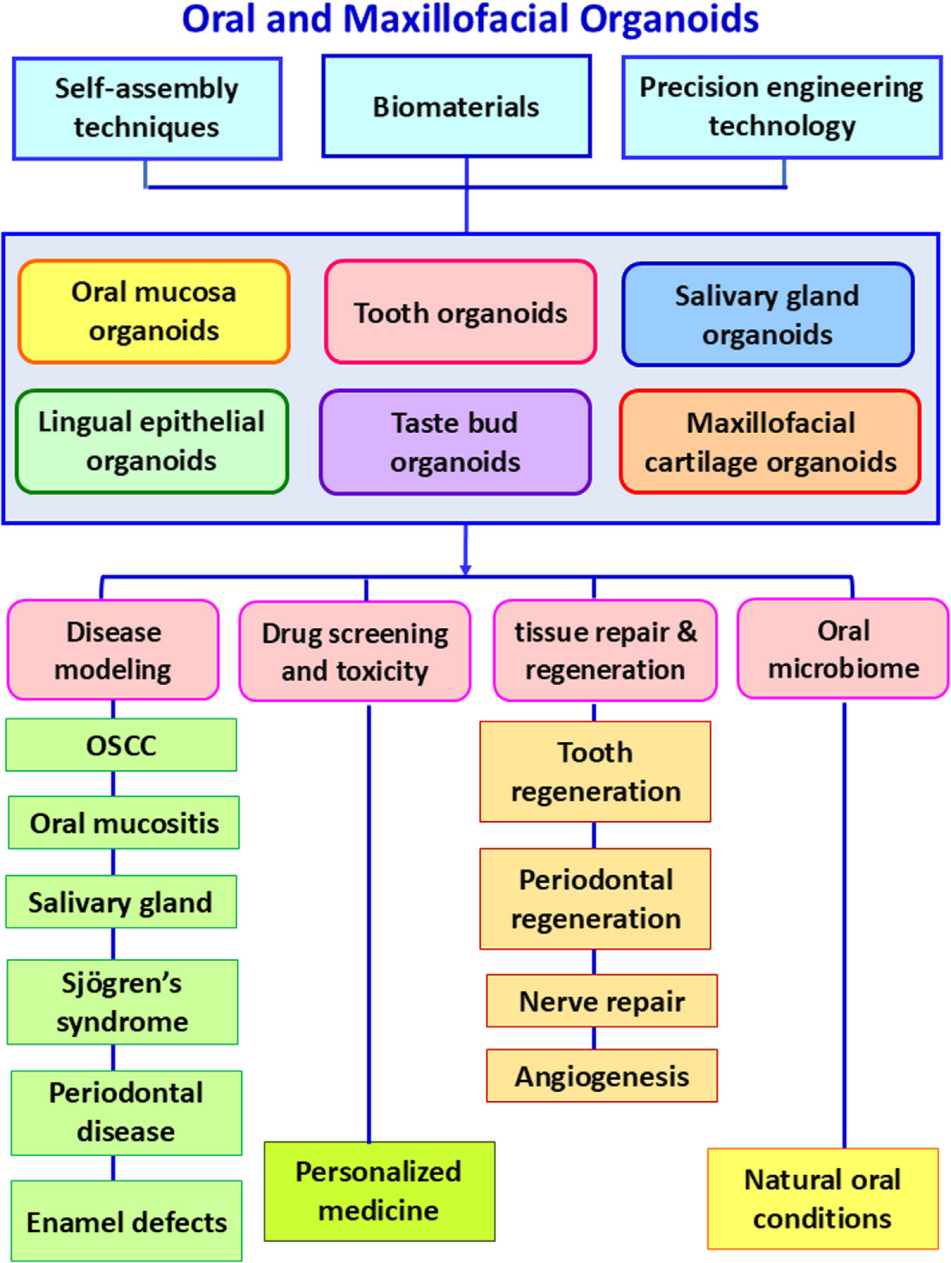
Applications of oral and maxillofacial organoids in dentistry and clinical research. Organoid systems are increasingly utilized for oral disease modelling, drug response, regenerative dentistry, and oral microbiome. These biomimetic three-dimensional platforms provide promising clinical tools for investigating oral pathophysiology and developing regenerative and precision-based dental therapies. OSCC, oral squamous cell carcinoma.

Oral and maxillofacial organoids have demonstrated promising applications in disease modelling, drug screening and toxicity, tissue regeneration, and oral microbiome. Figure 2 represents the applications of oral and maxillofacial organoids in dentistry and translational research.

### The application of organoids in disease modelling

#### Oral squamous cell carcinoma

OSCC, the most common malignancy of the oral cavity, has been increasingly investigated using advanced organoid models. Under coculture conditions incorporating stromal components, such as cancer-associated fibroblasts and immune cells, OSCC organoids showed promising translational potential to reflect key features of the tumour microenvironment.^20^

Culturing TSCC cells, the most common subtype of OSCC, on decellularized tongue ECM has led to the development of organoids that promise to preserve tumour heterogeneity and histopathological features.^22^ These tongue ECM-based models provide advanced platforms for TSCC research, offering valuable clinical systems for studying tumour biology and evaluating therapeutic strategies.

#### Oral mucositis

Oral mucositis (OM) is a common and debilitating complication of cancer therapy that severely impairs oral function, nutrition, and quality of life, and often limits treatment tolerance. An innovative chemotherapy-induced OM model has been developed using oral mucosal organoids derived from human epithelial cell lines cultured within fibroblast-embedded collagen matrices, followed by exposure to 5-fluorouracil to mimic chemotherapy-induced mucositis.^57^ This biomimetic model reproduces key pathological features of clinical OM, including reduced DNA synthesis, characteristic apoptotic patterns, and cytoplasmic vacuolization, providing a valuable platform for studying the disease mechanisms. In addition, organoid systems have been adapted to investigate radiation-induced damage, particularly the responses of epithelial stem cells to irradiation. These models offer important mechanistic insights into radiation-related complications such as taste dysfunction and mucosal ulceration, and also support the development of targeted protective strategies.^58^

#### Sjögren’s syndrome and salivary gland

Sjögren’s syndrome is a chronic autoimmune disease that primarily affects the progressive lymphocytic infiltration of the exocrine glands, tissue destruction, and salivary dysfunction, manifesting as severe xerostomia.^59^ Recent studies have introduced innovative models using salivary gland epithelial cells isolated from patients with xerostomia and labial salivary gland biopsy specimens. These models provide a valuable platform for studying the disease mechanisms and screening potential therapeutic methods.^60^ Culturing human salivary gland cells within bioactive scaffolds has further enhanced organoid formation. In particular, collagen-cultured organoids derived from human salivary gland stem cells support allogeneic transplantation and represent an important step towards clinical application.^61^

Current research also focuses on understanding how the microenvironment regulates epithelial stem and progenitor cells during salivary gland development for xerostomia modelling.^62^

#### Periodontal disease

Periodontitis, the leading cause of tooth loss in adults, is characterized by progressive degradation of the periodontal ligament (PDL) and alveolar bone resorption. Advanced bioink-based models have recently been developed to study periodontal pathology by recapitulating selected features of the periodontal microenvironment. These systems effectively reproduce the composite anatomy of the PDL and adjacent alveolar bone.^63^ Notable progress in biomaterial design has led to the creation of composite scaffolds that combine hydrogels with nanoparticles, offering promising biocompatibility and causing mechanical properties similar to native periodontal tissues. In parallel, scaffold-free organoid models based on immortalized PDL fibroblast cell lines have been developed. These models show features associated with dental follicle cell differentiation and provide *in vitro* platforms for investigating functional PDL regeneration.^64^

Advanced organoid technologies that combine cellular components, bioactive scaffolds, and physiologically relevant mechanical stimuli have provided new insights into the molecular mechanisms underlying periodontal tissue destruction.^32^

#### Enamel defects

A pioneering mesenchymal-epithelial organoid model derived from dental follicle tissue can induce epithelial stem cell differentiation into functional ameloblasts.^65^ This system offers a valuable platform for exploring the diverse causes of enamel defects, including hereditary disorders such as amelogenesis imperfecta, bacterial infections, and various genetic mutations. Furthermore, an advanced 3D bilayer organoid model has been developed that combines dental pulp-derived ameloblast-like cells with odontoblasts to more accurately replicate the reciprocal epithelial-mesenchymal signalling characteristic of odontogenesis.^66^ Such models are suitable for studying both normal tooth morphogenesis and disease-related processes, enabling detailed investigation of enamel and dentin formation as well as diagnostic assessment of rare dental disorders such as amelogenesis imperfecta and dentin dysplasia.

### The application of organoids in drug screening and drug toxicity assessments

Oral organoids provide experimentally relevant systems for pharmaceutical development under physiologically relevant conditions. They can be used to evaluate mucosal anti-inflammatory compounds, anticancer agents, and dental biomaterials.^20^ In oncology, head and neck squamous cell carcinoma organoid biobanks have proven effective for assessing responses to conventional chemotherapeutics, such as cisplatin and carboplatin, as well as targeted therapies like cetuximab. Importantly, these biobanks enable robust correlations between genomic profiles and phenotypic drug sensitivity.^67^ This approach has particular relevance in personalized medicine, where patient-derived OSCC organoids generated from surgical specimens retain the original tumour’s genomic characteristics while revealing individual therapeutic susceptibilities through drug sensitivity testing. In oral and maxillofacial research, combining artificial intelligence (AI) with organoid-based systems enhances the accuracy and efficiency of screening. This underscores the importance of establishing disease-specific oral organoid biobanks for large-scale precision medicine research.^68^ Furthermore, organoid cultures derived from circulating tumour cells in patients with head and neck cancer present several advantages, including stable gene expression, patient specificity, and suitability for high-throughput drug screening. These models enable real-time assessment and improve the prediction of treatment response and drug resistance for patients.^69^

### The application of organoids in tissue repair and regeneration

3D oral organoid models are increasingly used in oral tissue repair and regeneration research, facilitating studies of stem cell biology, vascularization, and neural integration.^70^ On the other hand, the accessibility, high proliferative potential, and multilineage differentiation capacity of DPSCs, PDLSCs, and dental follicle cells make them highly valuable for orofacial reconstruction and dental tissue engineering.^19^ Furthermore, advances in biomaterials have enabled precise control of 3D tissue development. Modern bioprinting techniques allow spatially defined organization of cells and matrices, supporting the formation of complex tissue structures.^71^ Additionally, significant progress has been achieved in engineering vascular networks through coculture approaches. Vascularized dental pulp organoids have been successfully generated using DPSC-endothelial cell cocultures, while enhanced vascular endothelial growth factor expression in PDLSC-endothelial cell systems promotes robust angiogenesis.^72^ Finally, the generation of neuron-like cells from dental stem cells and their incorporation into 3D midbrain-like organoids provide new platforms for studying neurodegenerative mechanisms and modelling functional reinnervation in regenerated oral tissues.^73^ Together, these strategies present promising approaches for reconstructing complex orofacial defects and represent an emerging paradigm in regenerative dental medicine.

### The application of organoids in oral microbiome research

3D oral organoids enable controlled studies of host-microbiome interactions, allowing researchers to examine how specific microbial species maintain oral health or contribute to diseases such as dental caries, periodontitis, and OSCC. Asymmetric gas coculture platforms create physiological oxygen gradients that preserve the viability of anaerobic bacteria while maintaining gingival epithelial barrier function, providing unique opportunities to study bacterial invasion and epithelial immune responses.^74^ More advanced bioreactor-integrated gingival organoids incorporate dynamic fluid flow, ionic composition, and mechanical forces that potentially mimic natural oral conditions. These systems support complex microbial communities and reproduce key homeostatic mechanisms of periodontal pockets, including barrier maintenance and microbial balance.^75^ Oral microbiome research has thus shifted from focusing on individual pathogens to exploring complex microbial ecosystems and their systemic effects.

## Discussion

Compared with earlier descriptive reviews, the present work provides a more clinically oriented and translational comparison of oral and maxillofacial organoids, with emphasis on their relative readiness for regenerative and precision dentistry. OoC technologies in the dental, oral, and craniofacial field continue to advance towards recapitulating human immune and multiorgan functions. Therefore, these may help reduce dependence on animal models in selected preclinical applications for efficient therapeutic validation and clinical translation.^76^ Capturing the complex interactions between organs requires integrated platforms, which can be achieved by combining organoids with microfluidic technologies to create OoC devices and by linking multiple chips.^77^ Together, these innovations based on ‘human-on-a-chip’ platforms represent important advances towards the reliable, large-scale generation of physiologically relevant oral and maxillofacial organoids for both research and clinical applications.^78^ An organ-on-chip is a microengineered *in vitro* platform that recapitulates the key structural and functional features of a single organ or tissue. In contrast, a human-on-chip integrates multiple organ-on-chip systems through interconnected fluidic networks to model interorgan interactions and systemic physiological responses.^77^^,^^78^

Although there are considerable advantages to the application of organoid technology in dentistry and oral and maxillofacial research, challenges remain in some areas, including the need for scalability and standardization of oral and maxillofacial organoid production, establishing standardized protocols, and designing an appropriate composite of biomaterials. In addition, genomic instability and economic issues arise from long-term culture. Moreover, ethical concerns about the structure of organoids still pose challenges to scientists.^19^ Here, we discuss all the mentioned challenges in detail:

Both natural and synthetic scaffold materials offer notable advantages in organoid construction for biomedical research and clinical translation. However, these biomaterials also pose challenges, such as interbatch variability and sterilization difficulties.^79^ For example, when using organoids for dental pulp regeneration, it is necessary to determine the optimal stem cell density and number of organoids. In addition, restricted nutrient supply, limited removal of metabolic waste, and the complex internal architecture of the constructs contribute to the size constraints observed in current pulp organoid models.^20^ The poor solubility and limited long-term stability of some purified recombinant protein factors reduce the reproducibility required for the clinical translation of tumour organoids.^80^ Recent advancements in biomaterials through various cross-linking methods, including those activated by light, enzymes, or temperature, and the incorporation of different polymeric materials, have created new opportunities for organoid design and fabrication.^81^ For example, the application of a digital light processing system to create 3D bioprinting using bone marrow-derived mesenchymal stem cells was successful for *in vivo* and *in vitro* construction of cartilage organoids.^82^

To address remaining challenges of scalability and standardization, automated systems that combine magnetic levitation with robotic integration have further expanded production capacity, reaching outputs of up to 2000 organoids per hour.^83^ The Magnetic 3D Levitation (M3DL) system, a scaffold-free and substrate-free culture approach, facilitates the assembly of primary salivary gland-derived cells and supports their production of ECM. In addition, the development of columnar and poured-plate platforms has improved flow rates during casting, promoted 3D cell growth and high-throughput culture, minimized cell death in central regions, and enhanced operational flexibility and user convenience.^84^

Another complexity of organoid technology is the significant economic challenges it presents. Although organoid-based screening costs only about 5% of equivalent animal studies, long-term culture requires expensive growth factors and specialized media, and large-scale use demands advanced automation systems, which further increase costs. Emerging bioreactor and automation platforms may help expand production capacity, but cost-effective manufacturing that maintains biosafety and functional integrity will require additional technological advances.^85^ Another concern of oral organoid technology is the genomic stability of organoids during extended culture. Long-term *in vitro* growth can introduce genetic changes that alter phenotypic accuracy, thereby reducing the reliability of these systems for disease modelling and drug screening. This issue highlights the need for enhanced culture methods and rigorous quality control to maintain the biological safety of organoid systems.^86^ Moreover, ethical concerns about the use of embryonic stem cells in organoid development highlight the importance of iPSCs as an alternative.

In drug screening and toxicity testing, technical barriers include establishing standardized organoid biobanking protocols and optimizing AI-assisted screening platforms.^19^ Although AI and machine learning potentially improved organoid research, limited model interpretability and the lack of standardized datasets limit the generalizability of models across laboratories and organoid types. Developing explainable AI systems is therefore essential to clarify decision-making processes and strengthen clinical confidence.^87^

## Current barriers and emerging solutions

One of the most persistent barriers to clinical translation is the limited vascularization of organoid systems, which continues to constrain their size, viability, and functional maturation. For instance, salivary gland organoids cultured in Matrigel can achieve a relatively organized structure *in vitro*, but often exhibit reduced functionality after transplantation, particularly due to insufficient vascular integration. To overcome this issue, recent studies have explored approaches such as endothelial cell coculture and the use of prevascularized scaffolds to encourage microvessel formation. Microfluidic platforms have also been introduced to provide controlled perfusion, thereby improving nutrient delivery and waste removal in organoids.^15^

Another important limitation is the absence of immune components in most current organoid models, as most oral diseases involve complex immune-tissue interactions. Initial efforts to incorporate immune cells into oral and maxillofacial organoids have yielded encouraging results, particularly for modelling inflammatory conditions and tumour microenvironments. But these approaches are still technically inefficient and not yet widely standardized.^29^

## Recent advances and unmet clinical needs in dentistry

One of the most significant developments has been the increasing use of patient-derived organoids, which recapitulate specific features of interpatient variability and the genetic and phenotypic characteristics of the original tissue. This shift has been particularly relevant in oral oncology, where organoid platforms are being explored for real-time drug sensitivity screening and individualized treatment planning.^20^ In parallel, advances in 3D bioprinting have also enabled the development of bioengineered models of multiple cell types and biomaterials, which are essential for reconstructing complex oral tissues.^88^

Despite these advances, several clinical needs in dentistry remain insufficiently addressed. For example, effective long-term treatments for salivary gland dysfunction, particularly in patients with radiation-induced damage or autoimmune conditions, are still lacking.^30^ Similarly, predictable regeneration of periodontal tissues and complete tooth structures remains a major challenge, as current therapies often fail to restore the full structural and functional integrity of these issues. In oral oncology, there is an ongoing need for reliable platforms that can guide personalized therapy and more accurately predict treatment response than existing models.^20^

## Conclusion

Oral and maxillofacial organoid technologies represent promising experimental platforms for advancing disease modelling, regenerative dentistry, drug screening, and precision-based therapeutic research. However, important technical, biological, and translational challenges remain. Among current models, oral cancer and salivary gland organoids demonstrate promising translational potential, given their functional validation and scalability. Together, the continued refinement of these technologies may contribute to future advances in precision dentistry and oral disease research while reducing dependence on conventional animal models.

## Data availability

Data sharing is not applicable to this article as no datasets were generated or analysed during the current study.

## Reporting sex- and gender-based analyses

For the scope of this review and based on the analysed literature, no significant sex- or gender-dependent differences were consistently observed that would alter the general conclusions regarding stem cell efficacy or mechanisms discussed herein. Therefore, a specific statement on sex/gender differences is deemed not applicable to the overall findings presented in this review.

## Declaration of generative AI and AI-assisted technologies in the writing process

Generative AI or other AI tools were not used in the preparation of the manuscript, data analysis, or any other aspect of the research process.

## Author contributions

Mahtab Mottaghi contributed to the writing of the manuscript – reviewing and editing, the collection, study concept and design, and investigation; Amir Attaran Khorasani collaborated in the drafting of the manuscript and created the figures; Shahrzad Samadian participated in the in the drafting of the manuscript and investigation; Alieh Farshbaf contributed to the writing of the manuscript – reviewing and editing; Mehran Mohareri contributed to the writing of the manuscript and designed the table; Farzaneh Ahrari assessed the review to evaluate the studies selected, read, wrote, revised, and approved the final manuscript as well as study supervision.

## Conflict of interest

The authors declare that they have no known competing financial interests or personal relationships that could have appeared to influence the work reported in this article.
